# Intramuscular Nerve Bundles Reflect TDP‐43 Pathology in the Medulla and Spinal Cord of ALS Patients

**DOI:** 10.1111/nan.70016

**Published:** 2025-04-07

**Authors:** Takashi Kurashige, Tomomi Murao, Yuhei Kanaya, Yoriko Dodo, Tomohito Sugiura, Kazuya Kuraoka, Tomohiko Ohshita

**Affiliations:** ^1^ Department of Neurology NHO Kure Medical Center and Chugoku Cancer Center Kure Japan; ^2^ Department of Diagnostic Pathology NHO Kure Medical Center and Chugoku Cancer Center Kure Japan

**Keywords:** amyotrophic lateral sclerosis (ALS), anterior horn, hypoglossal nuclei, intramuscular nerve bundles, transactive response DNA‐binding protein 43 (TDP‐43)

Amyotrophic lateral sclerosis (ALS) is a clinical diagnosis based on a history of progressive motor dysfunction with a combination of upper motor neuron (UMN) and lower motor neuron (LMN) signs [1, 2, 3]. Degeneration of neuromuscular junctions and axons is thought to be an important aspect of the pathogenesis of ALS. Recently, we have found axonal accumulation of transactive response DNA‐binding protein 43 (TDP‐43) in intramuscular nerve bundles in both autopsy‐confirmed ALS with TDP‐43 pathology (ALS‐TDP) and in patients diagnosed with ALS by muscle biopsy, suggesting this accumulation may be characteristic of patients with ALS [4]. However, despite the close correlation between the severity of TDP‐43 pathology and spinal motor neuron loss [5], the relationship between peripheral pathology and TDP‐43 pathology in the spinal cord is unclear.

We examined muscle, medulla and spinal cord tissue obtained from 11 patients with ALS‐TDP and 12 without ALS at the National Hospital Organization Kure Medical Center and Chugoku Cancer Center. Some of these patients were examined in our previous report [4]. None of the patients included in this study had a family history of ALS or other neuromuscular diseases, and none had pathological variants in the known causative genes of ALS. Additionally, none of the patients used tracheostomy‐based positive pressure ventilation, and they were confirmed to have the TDP‐43 pathology by post‐mortem examination. The distribution of TDP‐43 pathology was evaluated according to the method of Brettschneider et al. [6].

The study was approved by the institutional review boards of the National Hospital Organization Kure Medical Center and Chugoku Cancer Center (2020–15) and adhered to the Standards for Reporting of Diagnostic Accuracy (STARD) reporting guidelines. All examinations were performed after obtaining written informed consent from all participants or their families for diagnostic purposes followed by research application.

Muscle tissue of the tongue, diaphragm or rectus femoris obtained from ALS‐TDP patients were examined as either formalin‐fixed, paraffin‐embedded sections or frozen sections fixed by liquid nitrogen‐cooled isopentane as previously described [4]. Medulla and spinal cord tissue were prepared as 8‐μm‐thick formalin‐fixed, paraffin‐embedded sections and were examined by routine histopathology and immunohistochemistry. For intramuscular nerve bundles, we made 10 transverse step sections (8‐μm thick) at 50‐μm intervals to perform a thorough evaluation. These sections were immunostained using a Ventana BenchMark ULTRA automated slide staining system (Ventana Medical Systems) with a mouse monoclonal pTDP‐43 antibody (TIP‐PTD‐M01, pSer409/410, 1:3000 [CosmoBio, Tokyo, Japan]) and a rabbit monoclonal choline acetyltransferase (ChAT) antibody (ab178850, 1:500 [abcam, Cambridge, UK]) according to manufacturer instructions.

From spinal cord sections of ALS‐TDP patients, we evaluated the number of ChAT‐positive neurons in both hypoglossal nuclei (CNXII) (Figure S1A–F) and the anterior horns bilaterally (Figure S1G–L) at the C4 and L2 levels. We also counted pTDP‐43‐positive inclusions (Figure S1M,N). In intramuscular nerve bundles (Figure S1O), we detected bundles with axonal pTDP‐43 accumulation (Figure S1P), as we have previously reported [4]. Two observers (T.K., T.M.) individually accessed the histopathological findings of the medulla, spinal cord and skeletal muscle and, if required, a joint assessment was performed through scrutiny with a 2‐headed microscope. The myopathological findings were assessed blind to the neuropathological findings. Patient details are summarized in Table 1 and Table S1.

**TABLE 1:** Characteristics of ALS cases.

Case	1	2	3	4	5	6	7	8	9	10	11
Age (Y)	60s	80s	80s	70s	60s	80s	70s	70s	80s	70s	80s
Subtype	Bulbar	Flail arm	Flail arm	PMA	Flail arm	Bulbar	Bulbar	Bulbar	PMA	Bulbar	PMA
Duration (Mo)	6	6	7	14	15	16	24	32	110	19	24
Dysphagia	+	−	+	−	−	+	+	+	−	+	−
TDP‐43 pathology (Brettschneider stage)	2	2	4	3	3	4	4	4	2	2	4
Tongue inclusions/bundles (%)	26/41 (63.4)	33/56 (58.9)	18/42 (42.9)	39/56 (69.6)	27/57 (47.4)	41/51 (80.4)	32/37 (86.5)	36/51 (70.6)	5/12 (41.7)	23/38 (60.5)	18/34 (51.4)
CNXII											
Remained neurons	21	22	25	24	26	16	15	10	24	13	24
Neurons with inclusions (%)	16 (63.6)	14 (63.6)	2 (8.0)	5 (20.8)	10 (38.5)	11 (68.8)	5 (33.3)	4 (40.0)	16 (66.7)	10 (76.9)	15 (62.5)
Diaphragm inclusions/bundles (%)	43/68 (63.2)	51/70 (72.9)	60/71 (84.5)	37/51 (72.5)	34/66 (51.5)	45/55 (81.8)	45/67 (67.2)	34/44 (77.3)	7/11 (63.6)	14/22 (63.6)	22/41 (53.7)
C4											
Remained neurons	20	21	24	31	8	16	18	15	19	16	17
Neurons with inclusions (%)	6 (30.0)	17 (81.0)	21 (87.5)	20 (64.5)	1 (12.5)	12 (75.0)	11 (61.1)	8 (53.3)	10 (52.6)	11 (68.8)	9 (52.9)
Biceps inclusions/bundles (%)		21/25 (84.0)	11/15 (73.3)	16/26 (73.1)		17/22 (77.3)	16/23 (69.6)	12/14 (85.7)	2/2 (100.0)	17/22 (77.3)	19/24 (79.2)
C6											
Remained neurons		32	31	41		24	32	36	18	22	19
Neurons with inclusions (%)		15 (46.9)	22 (71.0)	30 (73.2)		15 (62.5)	21 (65.6)	28 (77.8)	16 (88.9)	16 (72.7)	14 (73.7)
Iliopsoas inclusions/bundles (%)	5/ 8 (62.5)	8/14 (62.5)	6/14 (42.9)	11/17 (64.7)	7/19 (36.8)	10/13 (76.9)	6/18 (33.3)	5/11 (45.5)	8/ 9 (88.9)	3/ 7 (42.9)	6/ 8 (75.0)
L2											
Remained neurons	29	25	30	12	24	30	26	28	8	31	16
Neurons with inclusions (%)	8 (27.6)	14 (56.0)	5 (16.7)	8 (66.7)	8 (33.3)	18 (60.0)	17 (65.4)	19 (67.9)	2 (25.0)	19 (61.3)	9 (56.3)

Abbreviations: CNXII, hypoglossal nuclei; F, female; M, male; Mo, Months; PMA, progressive muscular atrophy; Y, years.

We determined the number of remaining neurons, the percentage of intramuscular nerve bundles with axonal pTDP‐43 accumulation and the percentage of neurons with pTDP‐43‐positive and round inclusions in the CNXII of the medulla and the anterior horn of the spinal cord. All values are expressed as mean ± SD unless stated otherwise. We confirmed differences between means with the Kruskal‐Wallis test and correlations between nerve bundles and neurons or inclusions with Pearson's correlation coefficient using the Prism 8 software (GraphPad Software, La Jolla, CA). All *p*‐values were 2‐tailed, and a *p*‐value  <  0.05 was considered significant. The data for this study were collected in January 2024 and analysed in March 2024. The details of the analysis are described in Figure 1 and Tables S2 and S3.

**FIGURE 1:**
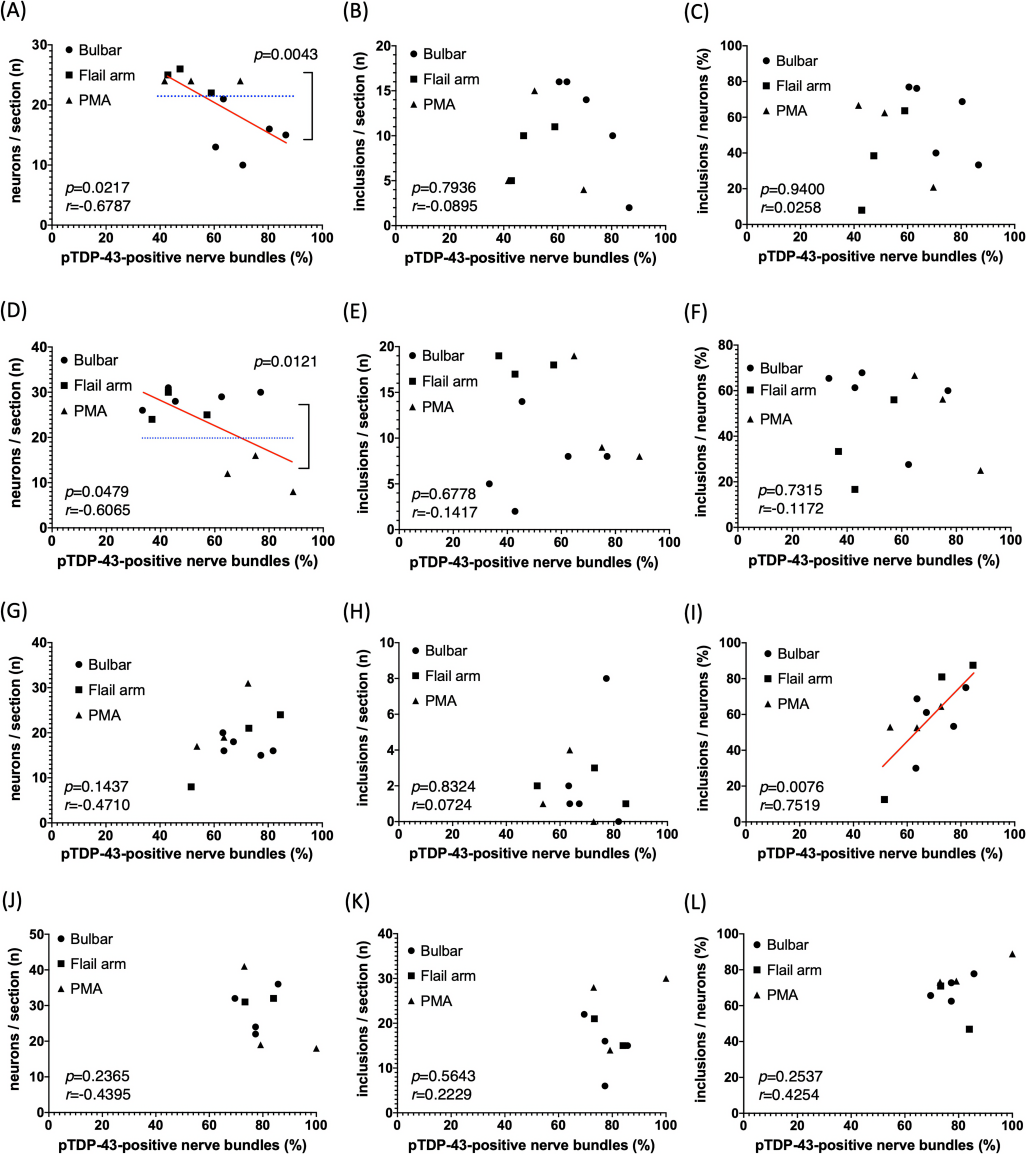
Correlations between intramuscular nerve bundles and the pathology in the central nervous system. (A) The percentage of axonal pTDP‐43‐positive bundles in the tongue was negatively correlated with remaining neurons in the hypoglossal nucleus (CNXII). (B, C) The percentage of axonal pTDP‐43‐positive bundles in the tongue was not correlated with the number (B) or the percentage (C) of remaining hypoglossal neurons with TDP‐43‐positive inclusions. (D) The percentage of axonal pTDP‐43‐positive bundles in the iliopsoas muscle was negatively correlated with remaining neurons in the anterior horn of L2. (E, F) The percentage of pTDP‐43‐positive bundles in the iliopsoas muscle was not correlated with the number (E) or the proportion (F) of remaining neurons with pTDP‐43‐positive inclusions in the anterior horn of L2. (G, H) The percentage of pTDP‐43‐positive bundles in the diaphragm was not correlated with the number of remaining neurons (G) or the number of remaining neurons with pTDP‐43‐positive inclusions (H) in the anterior horn of C4. (I) The percentage of pTDP‐43‐positive bundles in the diaphragm was positively correlated with the proportion of remaining neurons with pTDP‐43‐positive inclusions in the anterior horn of C4. (J‐L) The percentage of axonal pTDP‐43‐positive bundles in the biceps brachii muscle was not correlated with the number of remaining neurons (J), the number of neurons with pTDP‐43‐positive inclusions (K), or the proportion of neurons with pTDP‐43 inclusions (L) in the anterior horn of C6.

The fraction of nerve bundles with axonal pTDP‐43 accumulation (61.2 ± 14.8%) in the tongue negatively correlated with the number of remaining neurons in CNXII (20.0 ± 5.5/ section) (Figure 1A, *p* = 0.0217, *r* = −0.6787). Greater numbers of surviving neurons were observed in CNXII in non‐bulbar‐form ALS patients than in bulbar‐form patients (*p* = 0.0043). In addition, the percentage of positive bundles was higher in bulbar‐form patients than in non‐bulbar‐form ALS (*p* = 0.0173). However, the percentage of bundles that were positive was not correlated with either the number of neurons with pTDP‐43‐positive inclusions (1.6 ± 1.4/section) (*p* = 0.7936, *r* = −0.0895) (Figure 1B) or the fraction that contained them (50.5 ± 23.5%) (*p* = 0.9400, *r* = 0.0258) (Figure 1C).

Examination of the iliopsoas muscle and the L2 anterior horn again showed that the percentage of nerve bundles with axonal pTDP‐43 accumulation (57.0 ± 18.2%) correlated with the number of remaining neurons (23.5 ± 7.9/ section) (Figure 1D, *p* = 0.0479, *r* = −0.6065). Fewer neurons remained in the L2 anterior horn in progressive muscular atrophy (PMA)‐form ALS patients than in non‐PMA‐form patients (*p* = 0.0121). The percentage of positive bundles was higher in PMA‐form patients than in non‐PMA‐form patients (*p* = 0.0485). The percentage of positive bundles did not correlate with either the number of neurons with pTDP‐43‐positive inclusions (11.5 ± 6.1/section) (*p* = 0.6778, *r* = −0.1417) (Figure 1E) or the fraction with pTDP‐43‐positive inclusions (48.7 ± 19.1%) (Figure 1F, *p* = 0.7315, *r* = −0.1172).

The percentage of nerve bundles in the diaphragm with axonal pTDP‐43 accumulation (68.4 ± 10.6%) was not correlated with the number of remaining neurons in the anterior horn at C4 (18.6 ± 5.8/ section) (*p* = 0.1437, *r* = −0.4710) (Figure 1G) or the number of neurons with pTDP‐43‐positive inclusions (11.5 ± 6.0/ section) (*p* = 0.8324, *r* = 0.0724) (Figure 1H). However, it did correlate positively with the fraction of neurons with pTDP‐43‐positive inclusions (58.1 ± 21.9%) (*p* = 0.0076, *r* = 0.7519) (Figure 1I). ALS patients showed fewer than half the number of C4 anterior horn neurons as in control participants (39.3 ± 4.4/section), but the number of remaining neurons did not correlate with patients' clinical manifestation at onset (Figure 1G).

Biceps brachii muscle and C6 anterior horn showed no correlation between the percentage of nerve bundles with axonal pTDP‐43 accumulations (79.9 ± 9.1%) and the number of remaining neurons (25.9 ± 9.1/ section) (*p* = 0.2365, *r* = −0. 4395) (Figure 1J), the number of neurons with pTDP‐43‐positive inclusions (17.4 ± 7.4/section) (*p* = 0.8324, *r* = 0.0724) (Figure 1K) or the fraction of neurons with pTDP‐43‐positive inclusions (66.3 ± 13.9%) (*p* = 0.2537, *r* = 0.4254) (Figure 1L).

We further compared the percentage of pTDP‐43‐positive bundles with the severity of TDP‐43 pathology in the central nervous system as represented by the stages of pTDP‐43 pathology [6]. However, no significant associations were observed for any of the lesions examined in this study (Table S3).

TDP‐43 pathology severity closely correlated with motor neuron loss in the spinal cord, especially in the cervical anterior horn of flail‐arm‐form ALS patients and the lumbar anterior horn of PMA‐form patients [5]. In addition, the percentage of lingual intramuscular nerve bundles with axonal pTDP‐43 accumulation was negatively correlated with the number of remaining neurons in CNXII. CNXII neuron loss was more severe in bulbar‐form ALS patients than in non‐bulbar‐form patients, similar to findings previously reported in the cervical anterior horn of flail‐arm‐form and the lumbar anterior horn of PMA‐form ALS patients [5]. A negative correlation was also observed for the percentage of positive nerve bundles in the iliopsoas muscle and the remaining neurons at L2 in the lumbar spinal cord. In addition, the percentage of bundles in the iliopsoas muscle correlated positively with the fraction of L2 anterior horn neurons containing pTDP‐43‐positive round inclusions. Collectively, these findings suggest that the percentage of bundles with axonal pTDP‐43 accumulation might reflect the severity of motor neuron loss.

In contrast to the tongue and iliopsoas muscle, no correlation was observed between the percentage of pTDP‐43‐positive nerve bundles in the diaphragm and remaining neurons in the C4 anterior horn, although the percentage of pTDP‐43‐positive nerve bundles did positively correlate with the fraction of anterior horn neurons containing pTDP‐43‐positive inclusions. Previous electrophysiological studies of ALS patients showed that the motor unit number estimate, which is considered to reflect the number of motor neurons, decreased to approximately 30%–50% of that of normal control participants [7, 8, 9, 10]. In our study, none of the enrolled ALS patients used tracheal positive pressure ventilation, which was the only common feature among the patients, and the number of remaining neurons in ALS patients was less than half of that in control participants. Our findings suggest that loss of C4 motor neurons may cause respiratory failure or pneumonia, leading to death in ALS patients.

Our case‐control study suggests that the percentage of intramuscular nerve bundles with axonal pTDP‐43 accumulations might reflect the severity of motor neuron loss in ALS‐TDP patients. In addition, motor neuron loss in C4 may cause respiratory failure or pneumonia, leading to death. This is a single‐centre study which included preliminary results based on a few patients. Further prospective study of axonal pTDP‐43 accumulations in intramuscular nerve bundles is needed to understand the relationship of these accumulations with motor neuron loss in ALS patients.

## Author Contributions

T.K. conceived the study concept and design, acquisition, analysis, or interpretation of data and drafted the manuscript. T.M. reviewed clinical records, acquired data, and interpreted data. Y.K., Y.D., T.S., K.K. reviewed clinical records. T.O. reviewed the manuscript and supervised the study. All authors approved the final manuscript.

## Ethics Statement

The study was approved by the institutional review boards of the National Hospital Organization Kure Medical Center and Chugoku Cancer Center (2020–15) and adhered to the Standards for Reporting of Diagnostic Accuracy (STARD) reporting guidelines. All examinations were performed after having obtained written informed consent from all participants or their families for diagnostic purposes followed by research application.

## Conflicts of Interest

The authors declare no conflicts of interests.

## Supporting information


**Figure S1** Histopathology of lesions analysed in this study. (A, B) Hypoglossal nuclei and medulla of patients with sporadic amyotrophic lateral sclerosis (ALS). (C) Choline acetyltransferase (ChAT)‐positive neurons in the hypoglossal nuclei of ALS. (D, E) Hypoglossal nuclei and medulla of the control cases. (F) Choline acetyltransferase (ChAT)‐positive neurons in the hypoglossal nuclei of the control. (G, H) The anterior horn in the spinal cord of ALS. (I) ChAT‐positive neurons in the anterior horn. (J, K) The anterior horn in the spinal cord of the control. (L) ChAT‐positive neurons in the anterior horn of the control. (M) Phosphorylated transactive response DNA‐binding protein 43 (pTDP‐43)‐positive skein‐like inclusions in neurons. (N) pTDP‐43‐positive round inclusions in neurons. (O) Intramuscular nerve bundles. (P) Axonal pTDP‐43‐positive accumulations in intramuscular nerve bundles.

**Table S1** Characteristics of patients without ALS.

**Table S2** Kruskal–Wallis analysis.

**Table S3** Pearson correlation analysis between Brettschneider stage of TDP‐43 pathology and the percentage of pTDP‐43‐positive intramuscular nerve bundles.

## Data Availability

The data that support the findings of this study are available on request from the corresponding author. The data are not publicly available due to privacy or ethical restrictions.
