# Supplementary material for: Intramuscular Nerve Bundles Reflect TDP‐43 Pathology in the Medulla and Spinal Cord of ALS Patients
Source: Neuropathol Appl Neurobiol. 2025 Apr 7;51(2):e70016. doi: 10.1111/nan.70016 (PMC11974360; doi:10.1111/nan.70016)
Supplement: Supplementary file 1 — Figure S1 Histopathology of lesions analysed in this study. (A, B) Hypoglossal nuclei and medulla of patients with sporadic amyotrophic lateral sclerosis (ALS). (C) Choline acetyltransferase (ChAT)‐positive neurons in the hypoglossal nuclei of ALS. (D, E) Hypoglossal nuclei and medulla of the control cases. (F) Choline acetyltransferase (ChAT)‐positive neurons in the hypoglossal nuclei of the control. (G, H) The anterior horn in the spinal cord of ALS. (I) ChAT‐positive neurons in the anterior horn. (J, K) The anterior horn in the spinal cord of the control. (L) ChAT‐positive neurons in the anterior horn of the control. (M) Phosphorylated transactive response DNA‐binding protein 43 (pTDP‐43)‐positive skein‐like inclusions in neurons. (N) pTDP‐43‐positive round inclusions in neurons. (O) Intramuscular nerve bundles. (P) Axonal pTDP‐43‐positive accumulations in intramuscular nerve bundles. [file NAN-51-e70016-s002.docx]

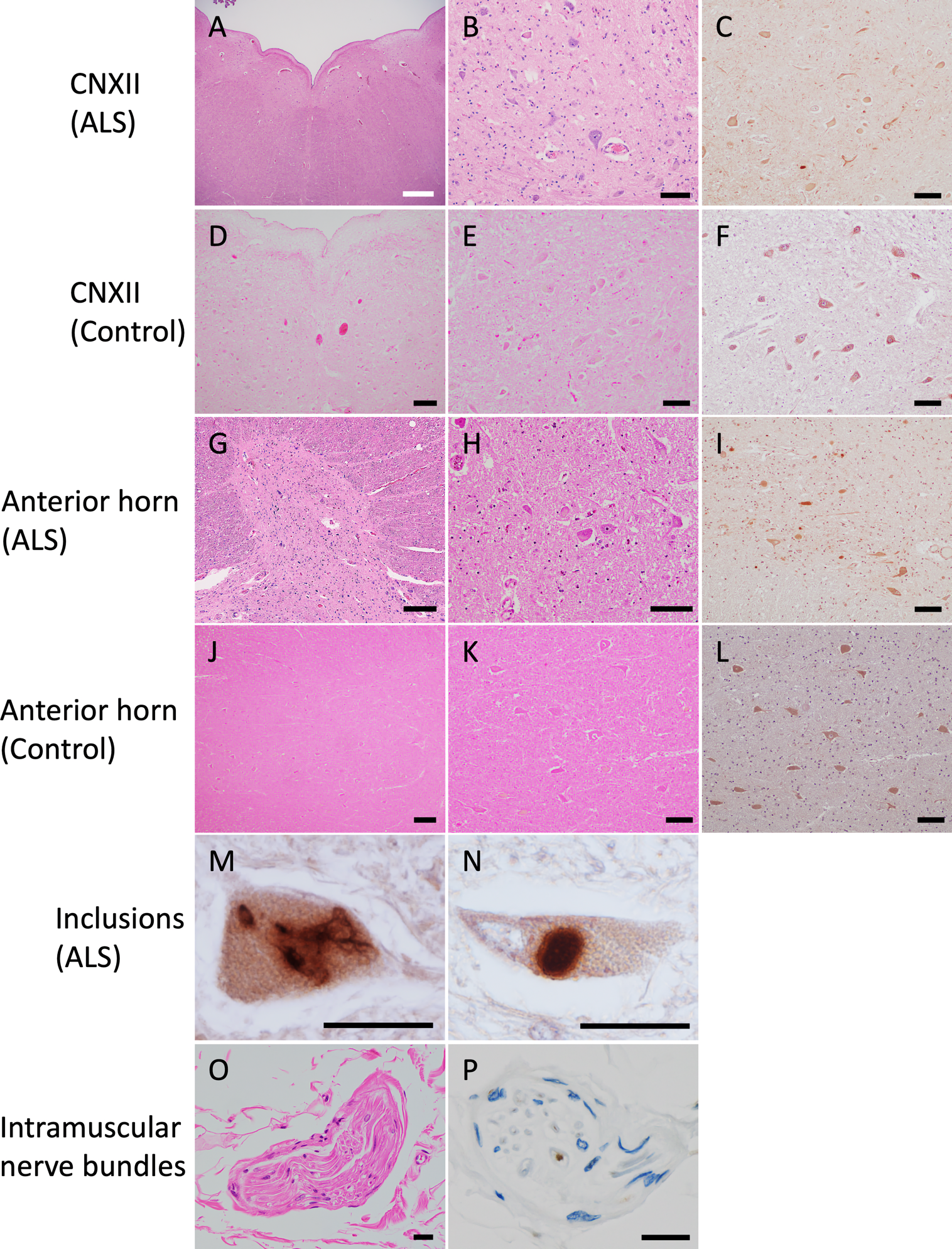


Supplemental Figure 1 Histopathology of lesions analysed in this study. (A, B) hypoglossal nuclei and medulla of patients with sporadic amyotrophic lateral sclerosis (ALS). (C) Choline acetyltransferase (ChAT)-positive neurons in hypoglossal nuclei of ALS. (D, E) hypoglossal nuclei and medulla of control cases. (F) Choline acetyltransferase (ChAT)-positive neurons in hypoglossal nuclei of control. (G, H) Anterior horn in the spinal cord of ALS. (I) ChAT-positive neurons in the anterior horn. (J, K) Anterior horn in the spinal cord of control. (L) ChAT-positive neurons in the anterior horn of control. (M) phosphorylated transactive response DNA-binding protein 43 (pTDP-43)-positive skein-like inclusions in neurons. (N) pTDP-43-positive round inclusions in neurons. (O) Intramuscular nerve bundles. (P) Axonal pTDP-43-positive accumulations in intramuscular nerve bundles.

Scale Bars (A, D) 500 µm (B, C, F) 100 µm, (E) 200 µm, (G, H) 10 µm, (I, J) 20 µm
