# Supplementary material for: Intramuscular Nerve Bundles Reflect TDP‐43 Pathology in the Medulla and Spinal Cord of ALS Patients
Source: Neuropathol Appl Neurobiol. 2025 Apr 7;51(2):e70016. doi: 10.1111/nan.70016 (PMC11974360; doi:10.1111/nan.70016)
Supplement: Supplementary file 2 — Table S1 Characteristics of patients without ALS. [file NAN-51-e70016-s003.docx]

Table 1 Characteristics of Patients without ALS

| Case | Age  (Y) | Diagnosis | Dysphagia | Tongue | CNXII | Diaphragm | C4 | Iliopsoas | L2 |
| --- | --- | --- | --- | --- | --- | --- | --- | --- | --- |
|  |  |  |  | Bundles (n) | Remaining  Neurons  per section | Bundles (n) | Remaining  Neurons  per section | Bundles (n) | Remaining  Neurons  per section |
| 1 | 50s | neuropathy | + | 62 | 34 | 20 | 45 | 13 | 46 |
| 2 | 60s | normal (AMI) | - |  | 30 | 23 | 38 | 14 | 48 |
| 3 | 80s | PD | - | 41 | 32 | 36 | 41 | 12 | 42 |
| 4 | 80s | CNS lymphoma | - | 36 | 32 | 18 | 38 | 16 | 38 |
| 5 | 40s | SAH | - |  | 38 | 16 | 34 | 19 | 44 |
| 6 | 60s | PD | - | 47 | 37 | 28 | 36 | 13 | 43 |
| 7 | 60s | AQP4(+) NMOSD | + |  | 22 | 33 | 34 | 11 | 38 |
| 8 | 80s | Sub dural hematoma | - |  | 36 | 34 | 48 | 14 | 41 |
| 9 | 70s | Cerebral infarct | - |  | 33 | 25 | 44 | 9 | 43 |
| 10 | 70s | Cerebral infarct | - |  | 32 | 22 | 37 | 10 | 39 |
| 11 | 70s | CBD | - |  | 28 | 28 | 36 | 16 | 36 |
| 12 | 80s | PSP | - |  | 33 | 51 | 39 | 21 | 38 |

Abbreviations: Y, years; Mo, Months; CNXII, hypoglossal nuclei; M, male; F, female
