# Supplementary material for: Intramuscular Nerve Bundles Reflect TDP‐43 Pathology in the Medulla and Spinal Cord of ALS Patients
Source: Neuropathol Appl Neurobiol. 2025 Apr 7;51(2):e70016. doi: 10.1111/nan.70016 (PMC11974360; doi:10.1111/nan.70016)
Supplement: Supplementary file 3 — Table S2 Kruskal–Wallis analysis. [file NAN-51-e70016-s004.docx]

Supplementary Table 2 Kruskal-Wallis analysis

|  |  | Bulbar | Flail arm | PMA | P value |
| --- | --- | --- | --- | --- | --- |
| CNXII | Remaining neurons (/ section) | 15.0 ± 4.1 | 24.3 ± 2.1 | 24.0 ± 0.0 | 0.0043 |
|  | pTDP-43-positive inclusions (%) | 59.0 ± 20.8 | 36.7 ± 27.9 | 50.0 ± 25.3 | 0.3338 |
| C4 | Remaining neurons (/ section) | 17.0 ± 2.0 | 17.7 ± 8.5 | 22.3 ± 7.6 | 0.4439 |
|  | pTDP-43-positive inclusions (%) | 57.6 ± 17.5 | 60.3 ± 41.5 | 56.7 ± 6.8 | 0.6677 |
| C6 | Remaining neurons (/ section) | 28.5 ± 6.6 | 31.5 ± 0.7 | 26.0 ± 13.0 | 0.7643 |
|  | pTDP-43-positive inclusions (%) | 69.7 ± 6.9 | 58.9 ± 17.0 | 78.6 ± 8.9 | 0.1556 |
| L2 | Remaining neurons (/ section) | 28.8 ± 1.9 | 26.3 ± 3.2 | 12.0 ± 4.0 | 0.0121 |
|  | pTDP-43-positive inclusions (%) | 56.4 ± 16.4 | 35.3 ± 19.7 | 49.3 ± 21.7 | 0.2323 |
