# Supplementary material for: Intramuscular Nerve Bundles Reflect TDP‐43 Pathology in the Medulla and Spinal Cord of ALS Patients
Source: Neuropathol Appl Neurobiol. 2025 Apr 7;51(2):e70016. doi: 10.1111/nan.70016 (PMC11974360; doi:10.1111/nan.70016)
Supplement: Supplementary file 4 — Table S3 Pearson correlation analysis between Brettschneider stage of TDP‐43 pathology and the percentage of pTDP‐43‐positive intramuscular nerve bundles. [file NAN-51-e70016-s001.docx]

Supplementary Table 3 Pearson correlation analysis between Brettschneider stage of TDP-43 pathology and the percentage of pTDP-43-positive intramuscular nerve bundles

|  | P value | R value |
| --- | --- | --- |
| Tongue | 0.0544 | 0.5932 |
| Diaphragm | 0.9909 | 0.0039 |
| Biceps brachii muscle | 0.4111 | -0.3137 |
| Iliopsoas muscle | 0.9112 | -0.0382 |
